# Dwell time shaping in inverse treatment planning for cervical brachytherapy

**DOI:** 10.1016/j.phro.2024.100672

**Published:** 2024-11-10

**Authors:** Frida Dohlmar, Björn Morén, Michael Sandborg, Torbjörn Larsson, Åsa Carlsson Tedgren

**Affiliations:** a Medical Radiation Physics, Department of Health, Medicine and Caring Sciences, Linköping University, Linköping, Sweden; b Center for Medical Image Science and Visualization, CMIV, Linköping University; c Department of Mathematics, Linköping University, Linköping, Sweden; d Department of Medical Radiation Physics and Nuclear Medicine, Karolinska University Hospital, Stockholm, Sweden; e Department of Oncology Pathology, Karolinska Institute, Stockholm, Sweden

**Keywords:** Brachytherapy, Treatment planning, Cervical cancer, Optimisation, Inverse treatment planning

## Abstract

**Background and purpose:**

Manual treatment planning for cervical brachytherapy is a challenging task; therefore, we investigated a method for inverse treatment planning using pseudo-structures to control the dwell distribution. Our hypothesis was that this method could produce treatment plans with a pear-shaped dose distribution and a high central dose, that comply with clinical constraints.

**Materials and methods:**

Data from 16 previously treated patients were used to compare three treatment planning methods: i) manual, ii) straightforward inverse, and iii) inverse with pseudo-structures. The treatment plans were compared using dose-volume histogram parameters and by analysing the dwell times, and the distribution of total reference air-kerma (TRAK) in the different parts of the applicator. Methods were evaluated in one treatment planning system and verified in a second treatment planning system.

**Results:**

The median dose to 90 % of the clinical tumor volume was 7.6 Gy, 7.8 Gy and 8.1 Gy for manual, pseudo-structure and straightforward methods respectively. Distribution of TRAK for the different parts of the applicator for the three methods (manual, pseudo-structures, and straightforward), with combined intracavitary and interstitial treatments, were for vaginal part 39 %, 33 % and 15 %, for intra-uterine part 47 %, 50 % and 47 % and for interstitial part 13 %, 17 % and 38 % respectively. The results were similar in the second treatment planning system.

**Conclusion:**

The developed pseudo-structures worked as intended in shaping the dwell time distribution and in meeting the clinical constraints for both investigated treatment planning systems.

## Highlights

• Pseudo-structures successfully shaped the isodoses and lowered loading in needles.
• Comparisons included dwell times and total reference air kerma distributions.
• Pseudo-structure method worked as intended in two different planning systems.

## Graphical abstract

**Figure:**
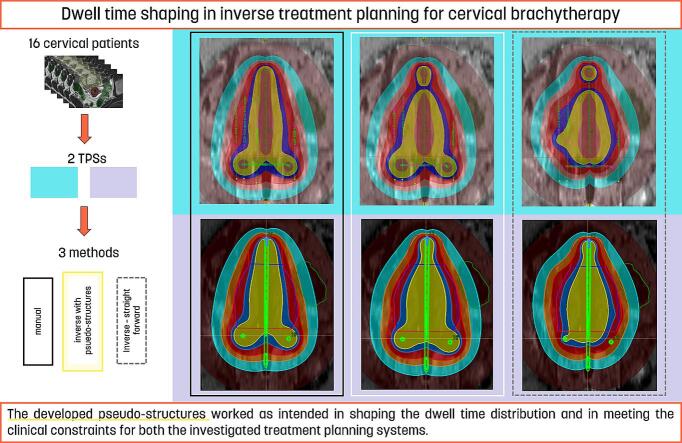

## 1 Introduction

Intracavitary (IC) or combined intracavitary/interstitial (ICIS) brachytherapy (BT) is an important and compulsory part of the curative treatment of advanced cervical cancer when surgery cannot be used. Treatment planning for cervical patients is a demanding task and is done under some stress for the treatment planner, as the patient and the anesthesia team are waiting. A more automated method could streamline the task and take off some burden from the treatment planner. Recent recommendations [1] on treatment planning of BT for cervical cancer emphasize the importance of the dwell time patterns; the goal is to give a high central dose, to avoid long dwell times in the interstitial (IS) part, and to preserve the pear-shape of the isodose. The pear-shape has for a long time been used and clinical studies have shown good results [2], which is the reason to maintain the pear-shape. The gold standard for using BT at this body site in today’s clinically available software is manual planning, as it provides the best control of the loading pattern of the different parts of the implant. The aim is to maintain a high central dose, including both IC and IS components. The contribution from the different parts is monitored through the total reference air-kerma (TRAK) [3].

The anatomy around the cervix makes the treatment challenging, as there are sensitive organs (for example bladder and bowel) surrounding the cervix and the uterus. Further, these organs are mobile and can change shape and location from imaging to treatment. It is important to consider the robustness of the treatment and avoid large high dose volumes near the target volume boundaries. A guide for the treatment planner is the evidence-based treatment planning aims by Tanderup et al. [4]. They divide the aims into hard and soft constraints, which are values for the dose-volume histogram (DVH) parameters for the target volumes and the organs at risk (OARs). There are also hard and soft constraints for dose points, defined in ICRU 89 [3]. The DVH parameters and dose points have a prioritising order to help in the decision making. The hard constraints should first be fulfilled and secondly, if possible, also the soft constraints.

Inverse planning is widely used clinically in prostate BT and the tools are often developed for this body site. There are several studies of the use of inverse planning for cervical cancer [5], [6], [7], [8], [9], [10], [11], [12], [13], [14]; some have used clinical available optimisation tools [8], [13], while for example Oud et al. [11] have used their own optimisation tool that is developed specifically for cervical cancer. Chajon et al. [10] stated that it is important to be careful when using inverse planning and to be aware of the risk of heterogeneities in the dose distribution. Inverse treatment plans often have less dwell positions in use, a larger number of long dwell times, and for cervical cancer treatments the pear-shaped isodoses are lost [10]. Pseudo-structures can be used to control the distribution of the dwell times [8], [10], [15] and to obtain the desirable pear-shaped dose distribution [5]. Despite the promising outcomes of these attempts the recommendations are still to use manual methods for treatment planning [1], [16].

Our hypothesis is that the use of well-selected pseudo-structures can enable the application of clinically available optimisation tools to plan brachytherapy for cervical cancer. The aim of this study was to design and evaluate a method that can effectively steer existing clinical optimisation tools towards meeting clinical constraints including the dwell-time requirements. The resulting method should be flexible enough to be automated in the available clinical treatment planning systems (TPSs), as it involves semi-automatically contoured pseudo-structures with origin in the applicator and contoured target.

## 2 Materials and methods

### 2.1 Patients

Data from the first BT fractions (fx) for 16 cervical cancer patients previously treated at the University Hospital in Linköping was used. The patients were treated using Varians 3D Interstitial Ring Applicator (GM11010190), consisting of a ring-part and an intrauterine tandem-part (IU). Six IC patients and ten combined ICIS patients were selected in consecutive chronological order, excluding patients with freehand placed needles. The patient cohort had an average volume of clinical target volume high risk (CTV-HR) of 49.6 cm3 (for IC 29.7 cm^3^ and for ICIS 61.5 cm^3^) ranging from 15 cm^3^ to 99.7 cm^3^, and an average volume of gross tumor volume (GTV) of 25.6 cm^3^ (for IC 14.1 cm^3^ and for ICIS 30.2 cm^3^) ranging from 5.2 cm^3^ to 55.2 cm^3^. GTV was not visible in two IC patients and therefore not contoured, and for one ICIS patient GTV and CTV-HR were contoured as the same volume. The ICIS patients had three to eight needles, with an average of six. All patients had rectum, sigmoid and bladder contoured, but some patients did not have a bowel contoured as this was too far away from the treated volume to risk high doses. The Swedish Ethical Review Authority has given consent to the use of anonymised data (EPM 2020–03331). All treatment planning was performed with an Ir-192 source, nominal source strength of 40.7 mGy/h. The voxel size of the final dose calculation for all treatment plans was set to a cube of 1 mm side length and calculated with TG43 formalism [17] in both TPSs.

### 2.2 Main treatment planning system

Manual treatment plans, axial-oblique MR T2 images and structure sets were exported anonymously from BrachyVision and then re-imported to BrachyVision.

The manual treatment plans were those originally used for the patients. The dwell positions were set with a step size of 5 mm, in the tandem from the tip until just above the ring dwell positions, and in the ring 5 mm from the tip and to the end (in total 17 positions). All dwell positions in IC were loaded with equal time and normalized to give 7 Gy to point A. Then the dwell positions in the IS part were added, starting from the tip going to the ring level with a step size of 5 mm. The dwell times were manually modified, not using any geometrical or graphical tools, starting with the IC part and adding the IS part when needed. The goal was to first fulfil the hard constraints in Tanderup et al. [4] and secondly, if possible, also the soft constraints. The treatment planning was done by a medical physicist and the final corrections were done in collaboration with a radiotherapist.

An inverse treatment plan was constructed for each patient case using structures contoured by the radiotherapist, this method was called the straightforward (SF) method. The dwell positions in the IC part were set as for the manual treatment plan. Dwell positions in the needles ranged from the top of the needle to slightly above the ring channel. All dwell times were set to 1 s before opening the inverse optimisation tool, VEGO TG-43 Volume Optimizer v 16.1 (Varian Medical systems Inc. Palo Alto, USA). The objectives were imported into the optimizer through a template and the volumes were corrected according to the objectives used, see Table 1. In the optimisation tool, a maximum dwell time was set to the maximal time used in any clinical treatment plan.

**Table 1:** The optimisation objectives used in BrachyVision. The absolute volumes had to be changed manually for each patient when the template was imported. Objectives for normal tissue and ring-structure were only used for the pseudo-structure method.

Structure	Limit	Volume	Dose (Gy)	Priority
GTV	Lower	98 %	7.74	200
GTV	Lower	98 %	8.31	100
CTV-HR	Lower	90 %	7.14	400
CTV-HR	Lower	98 %	5.83	200
CTV-HR	Lower	90 %	7.74	100
CTV-HR	Lower	98 %	6.50	100
Bladder	Upper	2 cm^3^	5.88	200
Bowel	Upper	2 cm^3^	4.98	100
Rectum	Upper	2 cm^3^	4.98	200
Sigmoid	Upper	2 cm^3^	4.98	100
Normal tissue	Upper	50 %	6.00	100
Normal tissue	Upper	2 cm^3^	7.75	100
Ring-structure	Lower	30 %	7.00	100

Alternative inverse treatment plans were constructed using both the structures contoured by the radiotherapist and two structures constructed to steer the dwell time pattern, this method is called the pseudo-structure (PS) method. It was developed on a separate patient cohort, see description in supplementary data and Figures S1-S2. The separate cohort was used to avoid overfitting of the method. Different structures were tested with the aim of achieving a treatment plan that fulfils the hard constraints and, if possible, also the soft constraints. The pseudo-structures should also support obtaining dwell time patterns that mimic the patterns of manually constructed treatment plans.

**Fig. 1:**
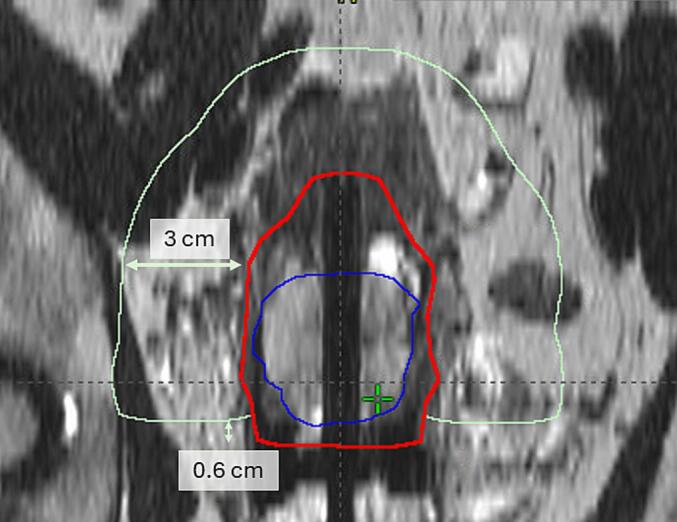

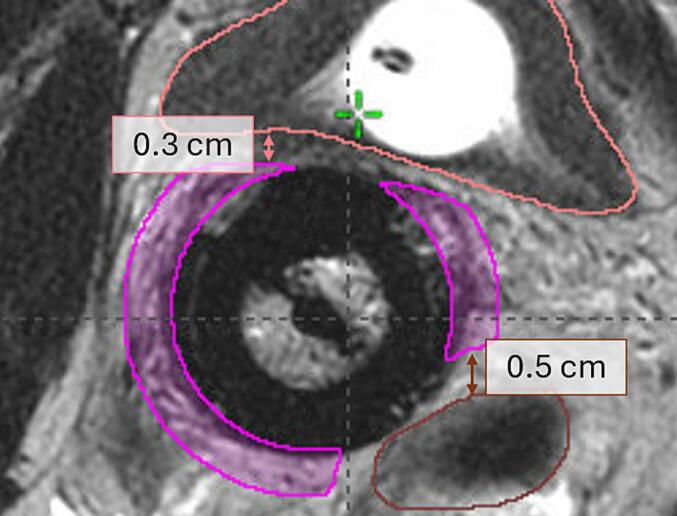
The pseudo-structures used for the pseudo-structure method: a) frontal view of the normal tissue structure (light green), and b) transversal view of the ring-structure (magenta). The other structures in the figures: CTV-HR (red), GTV (blue), bladder (pink), bowel (yellow), and rectum (brown).

Two pseudo-structures were created for each case, one normal tissue structure and one around the ring part of the IC, called ring-structure. The normal tissue structure was contoured as a 3 cm wide structure around CTV-HR, excluding the part adjacent to the ring-part of the IC, with a margin of two MRI-slices (i.e. 6 mm), see Fig. 1a. The aim of this PS was to reduce the dose to the surrounding healthy tissue, and for the ICIS patients also to reduce the dwell times in the needles. The resulting treatment plans should then have a high central dose and fewer hot spots near the boundaries of the target volume. The ring-structure was created as a 7 mm wide ring-structure around the outer part of the ring, excluding the part towards the CTV-HR. The structure was cropped to not include the OARs and a margin was left between the structure and the OAR, see Fig. 1b. The aim of the ring-structure was to obtain a more pear-shaped dose-distribution, to increase the TRAK contribution from the ring-part of the IC. The PSs were created semi-automatically using the tools in BrachyVision (Module Contouring). This process was timed for all patient cases, contouring and time spent in VEGO separately.

### 2.3 Alternative treatment planning system

To assess the generalisability of the method it was tested in another TPS. The anonymous manual treatment plans and the MR images and structure sets were exported from BrachyVision and imported to RayStation v. 2023B Research license (RaySearch Laboratories AB, Stockholm, Sweden). The import included the pseudo-structures.

No manual treatment planning was performed in RayStation. The manual treatment plans from BrachyVision were only recalculated in RayStation using the same dwell positions and dwell times.

The dwell positions were set as for the inverse treatment plans in BrachyVision. The ring-structure had to be changed to a target structure to be able to increase the dose in this volume. An external structure was created for each patient. It was manually contoured as the whole image set, and the material was set to water to be able to calculate dose. Optimisation objectives were imported through a template. Two inverse treatment plans were created, one using only the anatomical structures, called SF method, and one using both anatomical structures and the PS used in BrachyVision, called PS method. All dwell positions were reset before the optimisation was started and the maximum dwell time was set as in BrachyVision. In the Optimization settings the Optimization tolerance was set to zero and the max number of iterations was set to 500.

### 2.4 Plan comparison

The treatment plans were compared using established DVH parameters, which were taken from each system. Treatment plans in BT have different CTV-HR D_90%_ doses, as we aim to give at least the soft constraint. To be able to compare the treatment plans even though the target dose differ between the methods a sparing factor was calculated. The sparing factor was defined as the OAR dose (D_2cm3_) divided by the CTV HR D_90%_ and was calculated for all OARs.

The dwell time distribution was compared using the TRAK in the different parts of the applicator: ring, IU and IS. The incidences of long dwell times in the needles were analysed by the number of dwell times longer than 5 s, 10 s and 15 s, respectively.

### 2.5 Statistical analysis

The distributions of the data were analysed using the Shapiro-Wilk test for normality. The normally distributed data were statistically tested using one-way ANOVA with repeated measurement. Otherwise, the Friedman test and post hoc test were used. Bonferroni correction for multiple testing was included. The statistical analysis was performed in SPSS Statistics v. 29 (IBM, Armonk, New York). The significance level α was set to 0.05.

## 3 Results

### 3.1 Main treatment planning system

The time used to contour the PS in the main TPS was on average 2 min 49 s (± 40 s) and the time spent in the optimisation tool was on average 1 min 22 s (± 19 s).

**Fig. 2:**
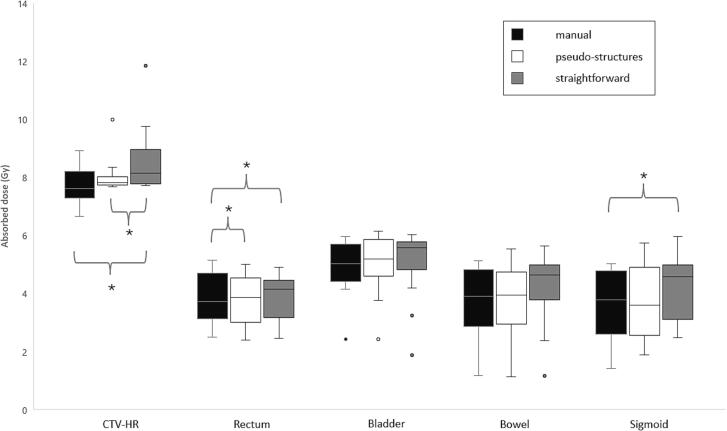
DVH parameters CTV-HR D_90%_ and for organs at risk D_2cm3_. The line marks the median, the boxes span between the lower quartile value and the upper, the whiskers mark the minimum data value and the maximum, and the points are the outliers. The comparisons marked with * were significantly different using Friedman’s test.

The DVH parameters showed few significant differences between the treatment planning methods, see Table 2 and Fig. 2. The median CTV D_90%_ was however higher for the inverse treatment plans, on average 0.2 Gy and 0.5 Gy for PS and SF respectively, but only significant change between manual and SF. Rectum median D_2cm3_ differed significantly between the manual inverse treatment plans, 0.2 Gy lower for PS and 0.4 Gy for SF. The sparing factor only showed significant changes for rectum for manual compared to SF, where the average was 0.04 lower for the SF method, see Table 2 and Figure S3.

**Table 2:** Plan comparison data for 16 treatment plans, except for relative TRAK contribution where IC (6 treatment plans) and ICIS (10 treatment plans) were evaluated separately. The median and the range (in parenthesis) were presented and Friedmans test was used except for TRAK contribution and the sparing factors where the average ± one standard deviation and oneway ANOVA repeated measurements was used.

	Manual	PS	SF	p-value Manual vs PS	p-value Manual vs SF	p-value PS vs SF
CTV D_90%_ (Gy)	7.6 (2.3)	7.8 (2.3)	8.1 (4.1)	n.s.	p < 0.01	0.04
CTV D_98%_ (Gy)	6.5 (4.6)	6.7 (3.3)	6.7 (2.6)	n.s.	n.s.	n.s.
GTV D_98%_ (Gy)	7.9 (7.1)	8.4 (7.8)	8.5 (8.1)	0.01	p < 0.01	n.s.
Rectum D_2cm3_ (Gy)	3.7 (2.7)	3.9 (2.6)	4.1 (2.5)	0.01	p < 0.01	n.s.
Bladder D_2cm3_ (Gy)	5.0 (3.5)	5.2 (3.7)	5.6 (4.2)	n.s.	n.s.	n.s.
Bowel D2_cm3_ (Gy)	3.9 (4.0)	3.9 (4.4)	4.6 (4.5)	n.s.	0.07	n.s.
Sigmoid D_2cm3_ (Gy)	3.8 (3.6)	3.6 (3.9)	4.6 (3.5)	n.s.	p < 0.01	n.s.
Max dwell time (s)	30 (37)	47 (25)	55 (37.9)	0.02	p < 0.01	n.s.
TRAK (cGy at 1 m)	4.4 (3.2)	4.2 (2.9)	4.4 (3.4)	n.s.	n.s.	n.s.
TRAK contribution vaginal (%)* IC	55 ± 13	57 ± 10	33 ± 24	n.s.	n.s.	0.08
TRAK contribution intra uterine (%)* IC	45 ± 13	43 ± 10	67 ± 24	n.s.	n.s.	0.08
TRAK contribution vaginal (%)* ICIS	39 ± 8	33 ± 4	15 ± 5	0.06	p < 0.01	p < 0.01
TRAK contribution intra uterine (%)* ICIS	47 ± 8	50 ± 10	47 ± 16	n.s.	n.s.	n.s.
TRAK contribution needles (%)* ICIS	13 ± 8	17 ± 11	38 ± 16	n.s.	p < 0.01	p < 0.01
Sparing factor_rectum_	0.50 ± 0.13	0.47 ± 0.11	0.46 ± 0.10	n.s.	p < 0.01	n.s.
Sparing factor_bladder_	0.63 ± 0.14	0.63 ± 0.13	0.61 ± 0.15	n.s.	n.s.	n.s.
Sparing factor_bowel_	0.49 ± 0.16	0.48 ± 0.16	0.50 ± 0.16	n.s.	n.s.	n.s.
Sparing factor_sigmoid_	0.48 ± 0.16	0.48 ± 0.17	0.50 ± 0.15	n.s.	n.s.	n.s.

PS – inverse pseudo-structure, and SF – inverse straightforward. TRAK – Total reference air-kerma. IC – intracavitary, ICIS – combined intracavitary and interstitial.

* Relative TRAK contribution from the different parts of the IC and ICIS.

n.s.- not significant (p > 0.1).

**Fig. 3:**
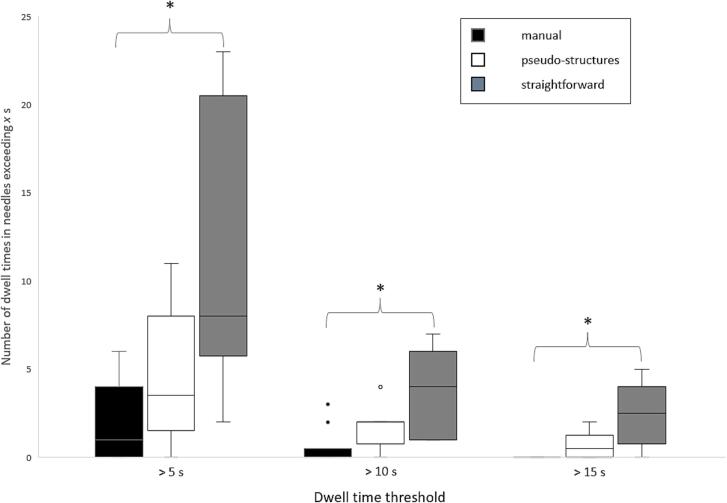
The number of dwell times exceeding 5 s, 10 s and 15 s in the needles. The line marks the median, the boxes span between the lower quartile value and the upper, the whiskers mark the minimum data value and the maximum, and the points are the outliers. The comparisons marked with * were significantly different using Friedman’s test.

The TRAK distribution showed no significant changes for the IC treatment plans, even though a larger standard deviation of the data could be seen for the SF plans 24% compared to manual 13% and PS 10%, see Table 2 and Figure S4. The TRAK distribution for ICIS cases showed that the vaginal part contribution was 24 percentage points lower for SF compared to the manual and the needle contribution were 25 percentage points lower for SF compared to manual. The result was confirmed by the number of dwell times in the IS exceeding 5 s, 10 s, and 15 s where significantly larger number of dwell times were found for SF compared to manual. The manual method had a median of 1 position exceeding 5 s and SF 8, see Fig. 3.

An example of the dose distribution for the three methods can be seen in Figure S5. Individual dwell times can be seen for one IC and one ICIS patient in Figure S6. It could be observed that less dwell positions were used in the inverse treatment plans.

### 3.2 Alternative treatment planning system

The comparison of DVH parameters between manual and PS, see Table S1, showed only significant changes for the median D_2cm3_ for bladder, where PS and SF had a 0.4 Gy and 0.5 Gy higher median dose than the manual. The result was confirmed by a 0.04 higher sparing factor for bladder. No other significant changes were found for the sparing factor. Examples of dose distribution for all three methods can be seen in Figure S7 and individual dwell times for one IC and one ICIS patient in Figure S8.

The TRAK distributions for the ICIS were comparable to the main TPS results. The IC TRAK distribution showed a significantly higher contribution from the vaginal part of 8 and 10 percentage points compared to manual and SF respectively. The number of long dwell times showed similar results in both systems, except for the dwell times exceeding 15 s, where no significant differences between the methods were observed in the alternative TPS, see Figure S9.

## 4 Discussion

Comparisons between three different treatment planning methods have shown that the use of pseudo-structures could help the inverse planning tool to produce treatment plans that resemble the manual gold standard method. This was done using an optimisation tool clinically available, but not developed for this body site, and tested in a second independent TPS showed similar results. The method could ease the implementation of ICIS cervical BT.

The use of pseudo-structures in inverse planning for cervical BT has previously been studied [5], [7], which inspired to some of the tested pseudo-structures. There have also been studies investigating commercially available optimisation tools. Trnková et al. showed results using a clinically available optimisation tool [6], [7]. This optimisation tool is developed for cervical BT and includes special features such as the possibility to have separate optimisation in the IC and the IS parts, respectively. They also conclude that an optimisation tool not designed for cervical BT needs to be combined with pseudo-structures to provide treatment plans with the desirable pear-shape. This work was done before the evidence-based dose planning aims [4] and the guidelines for cervical cancer management [1] were published. Therefore, a higher accepted dose to the bladder and no dose constraints on the bowel were used. Sharma et al. [5] also used a commercially available TPS and pseudo-structures, but they argued that the spatial distribution of the high dose volume are not important to preserve, and therefore their method was not designed to take this into account as our method do.

Our patient cohort includes some patients with large asymmetric tumors, which necessitates the use of large numbers of needles. The maximum dwell time was set high, in order to use the same through the whole cohort and for all three planning methods. If the method was used clinically, this could be set to a smaller value and in case of larger tumors, it could be changed accordingly in these rarer cases. The method could favourably be used as a first attempt and then the constraints and weight-factors could be altered to give individualized treatment plans. Swamidas et al. [15] used such a method to individualise the optimisation and it is a common method in external beam radiation therapy. Results showed that D_90%_ for CTV-HR is higher for the PS and SF (only significant changes for SF), but this is only a problem if the constraints for the OARs are not met. Rectum has a significantly higher dose for PS method compared to manual, but the average is still below the soft constraints from Tanderup et al. [4]. If it is too high, a new individualized inverse planning could be started with a higher weight factor for the rectum.

The study was performed on 16 patient cases, from one clinic, which is a rather small data set, but in the same order of magnitude as in other studies [5], [6], [7], [11]. One strength of this study is that the PS method is tested in two clinically available TPSs. Another strength of the PS method is that it is compatible and can be combined with other treatment planning tools, for example Multi-Criteria Optimisation (MCO), that is clinically used in external beam radiation therapy [18]. MCO has been tested in studies for cervical BT [11], [19], although not yet clinically available in the commercial TPSs.

We conclude that the developed pseudo-structures worked as intended in shaping the dwell time distribution and in meeting the clinical constraints. They can therefore be a useful tool in inverse treatment planning for cervical brachytherapy.

## Funding

This project was funded by LiU Cancer, Region Östergötland, the Swedish Cancer Foundation (CAN/2017/1029 and 211788 Pj) and the Swedish Research Council (VR-NT 2019-05416, VR-NT 2023-04181).

## Declaration of competing interest

The authors declare that they have no known competing financial interests or personal relationships that could have appeared to influence the work reported in this paper.
