# Supplementary material for: Dwell time shaping in inverse treatment planning for cervical brachytherapy
Source: Phys Imaging Radiat Oncol. 2024 Nov 10;32:100672. doi: 10.1016/j.phro.2024.100672 (PMC11605463; doi:10.1016/j.phro.2024.100672)
Supplement: Supplementary Data 1 [file mmc1.pdf]

Supplementary material

Contents

Overview study 1
Pseudo-structure method 1
Extra material – Results 4
References 10

Overview study

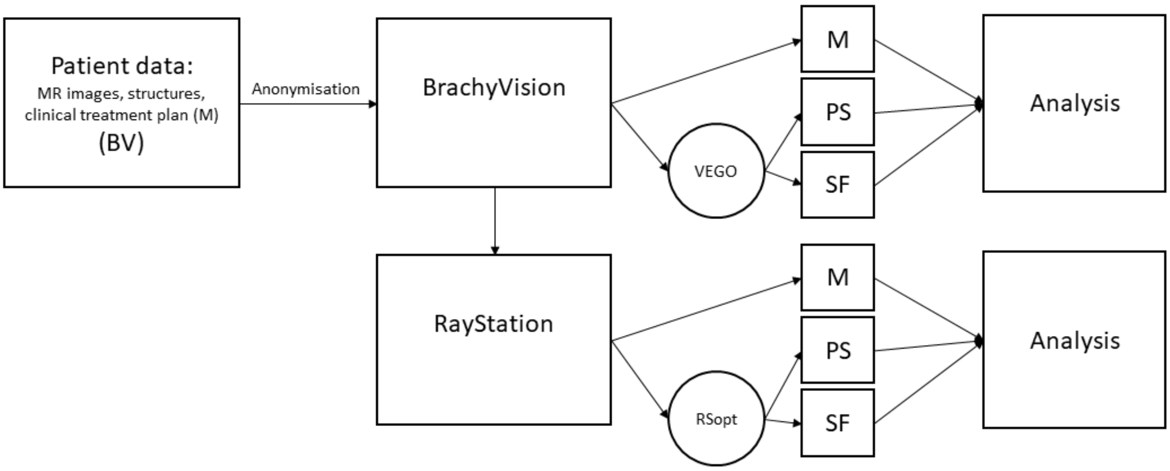

Figure S1: A flowchart of the study. VEGO is the name of the optimisation tool in BrachyVision and RSopt is an abbreviation of RayStation optimisation. M, PS and SF are three different treatment planning metods: M – Manual (no graphical or geometrical tools used), PS – inverse optimisation with target structures, organ-at-risk structures and the additional pseudo-structures, and SF – inverse optimisation with target structures and organ-at-risk structures (called straightforward method).

Pseudo-structure method

The treatment planning method using pseudo-structures (PS) was developed through systematically testing different types of pseudo-structures and tools available in the optimiser. All the tests were performed in the VEGO TG43 optimizer in BrachyVision v 16.1 (Varian Medical Systems Inc. Palo Alto, USA). VEGO was always started with 1 second in each active source position. The same objectives for the structures contoured by the oncologist were used with the straightforward method, see Table 1. For some tests upper constraints for high-risk clinical tumor volume (CTV-HR) (and also CTV-IU, see below) were added to the objectives in Table 1.

The tools tested were:

- Smooth (dwell time objective)
- Basal dose objective

The smoothing tool can be set between 0 and 300 and the tests were performed using 0, 97, 198 and 300. The smoothing tool was not used as it often gave a result with the same dwell time for all positions in one part of the applicator. Basal points were also excluded after some testing.

The pseudo-structures tested were:

- Normal tissue-structure
  A structure 3 cm wide around the CTV HR, starting 6 mm from the ring, see Figure 1a
- Ring-structure
  Structure around the ring part of the applicator excluding the part adjacent to CTV HR and the OARs. For the OARs the exclusion is done with some margin between the ring-structure and the OAR, ranging from 3 to 5 mm depending on the dose threshold, see Figure 1b. Trnková et al. [1] also used a structure around the ring-part, though they did not explain how was contoured.
- Inner control-structure
  A tube consisting of the outer part of CTV HR (5 mm broad). Starting 1 cm from the ring and excluding the top of the CTV HR, see Figure S2a.
- Standard loading-structure (pear-structure)
  A structure is created from the 100%-isodose of a standard loading treatment plan. This structure is combined with the CTV HR, see Figure S2b. Inspired by Sharma et al. [2] .
- Standard loading inner-structure
  A tube consisting of the outer part of standard loading structure (5 mm broad). Starting 1 cm from the ring and excluding the top of the CTV HR, see Figure S2c.
- Intra-uterine-structure
  A structure for the intra-uterine part of the applicator, only including the part inside CTV HR, starting 6 mm above the ring applicator, see Figure S2d.
- CTV-IU
  The intra-uterine-structure were removed from the CTV HR.

The different structures were tested on a set of patients, different to those used in the study. The patient cases consisted of three IC patients and seven ICIS patients. The volume of CTV-HR was on average 47.8 cm^3 (for IC 42.9 cm^3 and for ICIS 49.9 cm^3) ranging from 25.9 cm^3 to 69.9 cm^3. The volume of gross tumor volume (GTV) was on average16.7 cm^3 (for IC 15.4 cm^3 and for ICIS 16.7 cm^3) ranging from 6.4 cm^3 to 22.3 cm^3. The fulfillment of the soft and hard constraints were evaluated as well as the dwell time pattern in the different parts of the applicator. To get a quick overview of the results of the optimisation, the acceptance rate was used. This is defined as how many of the criteria in Tanderup et al. [3] that was fulfilled. The acceptance rate is described by Belanger et al. [4].

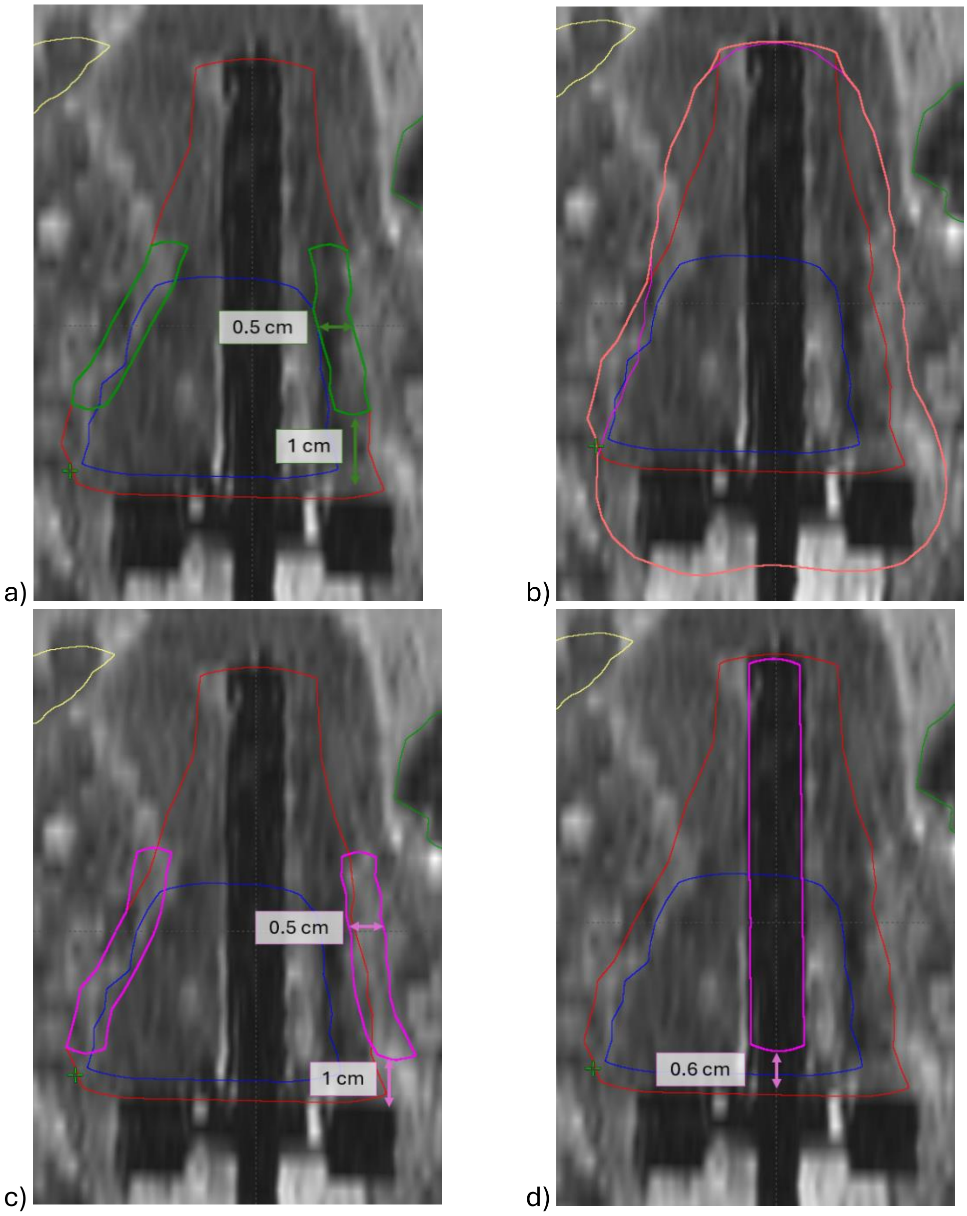

Figure S2: The pseudo-structures used to develop the pseudo-structure method, a) Inner control-structure, b) Standard loading-structure (pear-structure), c) Standard loading inner-structure, and d) Intra-uterine-structure.

Extra material- Results

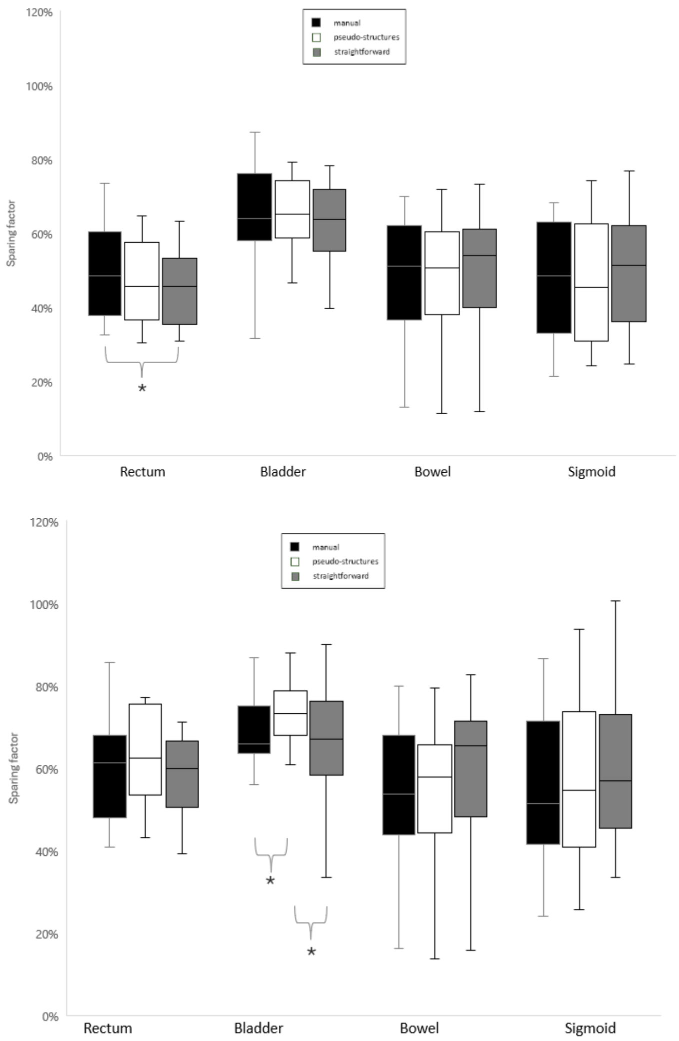

a)

b)

Figure S3: The sparing factor, OAR dose divided by the target dose (clinical target volume – high risk (CTV-HR), for a) main TPS, and b) alternative TPS. The line marks the median, the boxes span between the lower quartile value and the upper, the whiskers mark the minimum data value and the maximum, and the points are the outliers. The comparison marked with * were significantly different using one-way ANOVA repeated measurements.

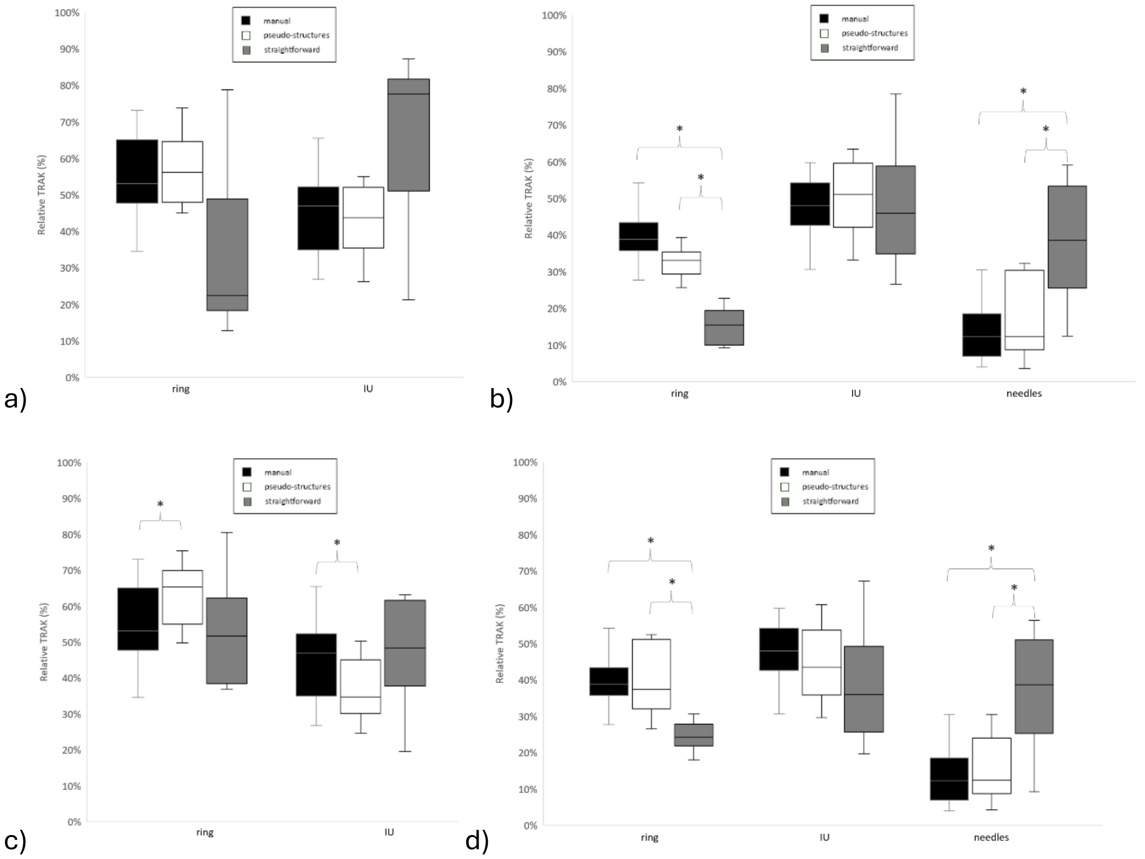

Figure S4: The relative TRAK for the different parts of the applicator: ring, intrauterine tandem (IU), and needles: a) Intracavitary main TPS (IC), b) combined IC and interstitiell (ICIS) main TPS, c) IC alternative TPS, and d) ICIS alternative TPS. The comparison marked with * are significantly different using one-way ANOVA repeated measurements.

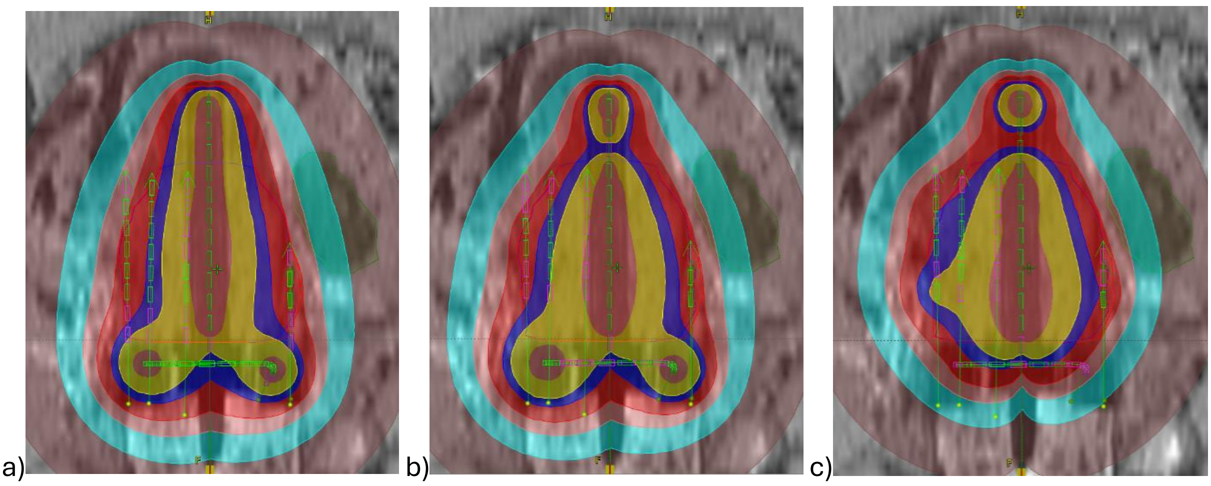

Figure S6: Frontal view of dose distribution for the three different treatment planning methods in main TPS, a) manual, b) pseudo-structure, and c) straightforward. Dose levels: yellow 14 Gy, blue 10.5 Gy, red 7 Gy, pink 5.25 Gy, turquoise 3.5 Gy, and brown 1.75 Gy. Structure contours are red CTV-HR, blue GTV, and green sigmoid.

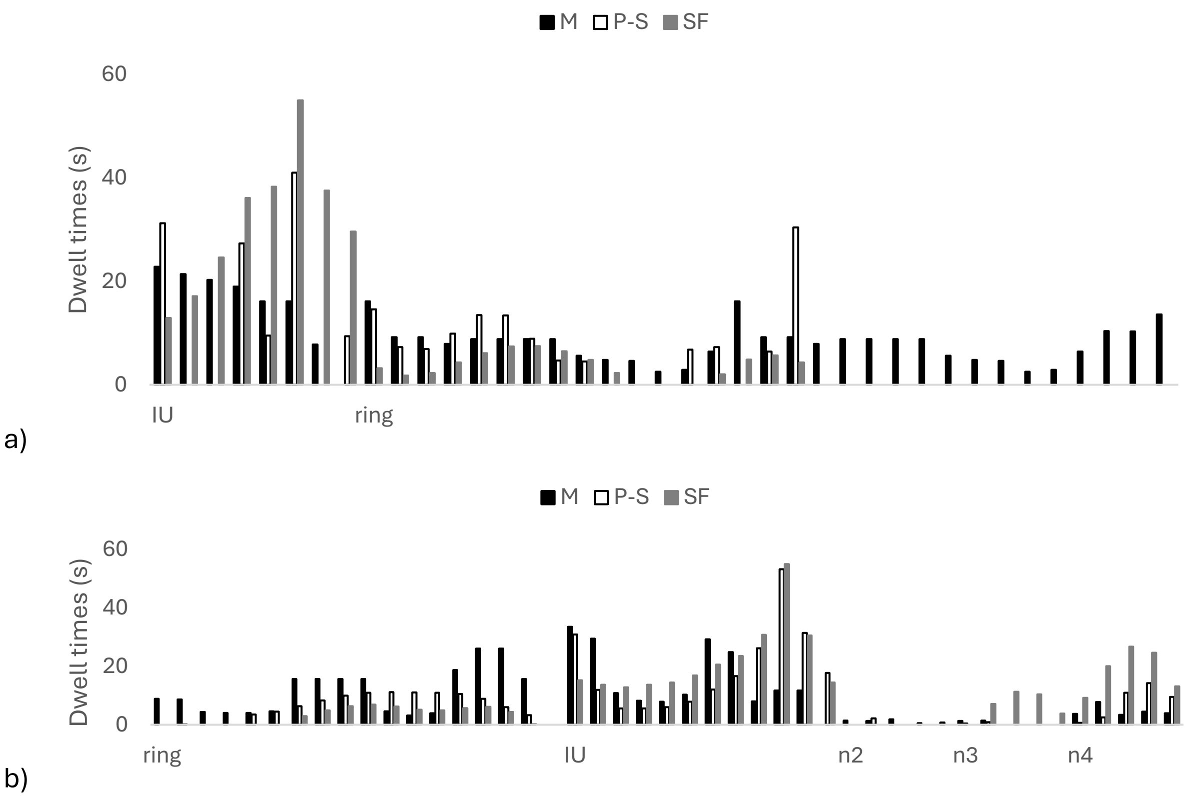

Figure S7: All dwell times from the tip of the applicator part going towards the afterloader for two main TPS patients: a) IC, and b) ICIS. Here the three methods are denoted: M – manual, P-S – pseudo-structure, and SF – straightforward.

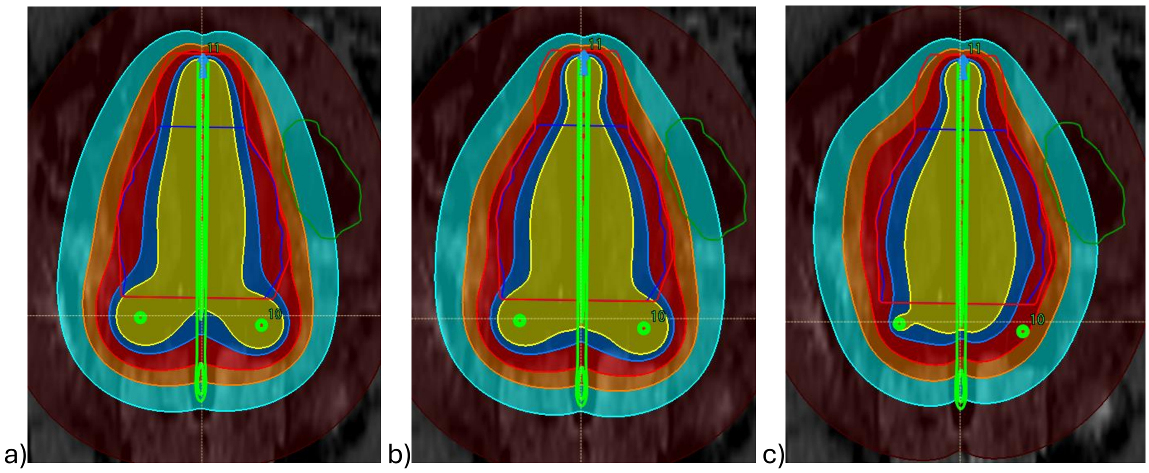

Figure S8: Frontal view of dose distribution for the three different treatment planning methods in alternative TPS, a) manual, b) pseudo-structure, and c) straightforward. Dose levels: yellow 14 Gy, blue 10.5 Gy, red 7 Gy, orange 5.25 Gy, turquoise 3.5 Gy and brown 1.75 Gy. Structure contours are red CTV-HR, blue GTV, and green sigmoid.

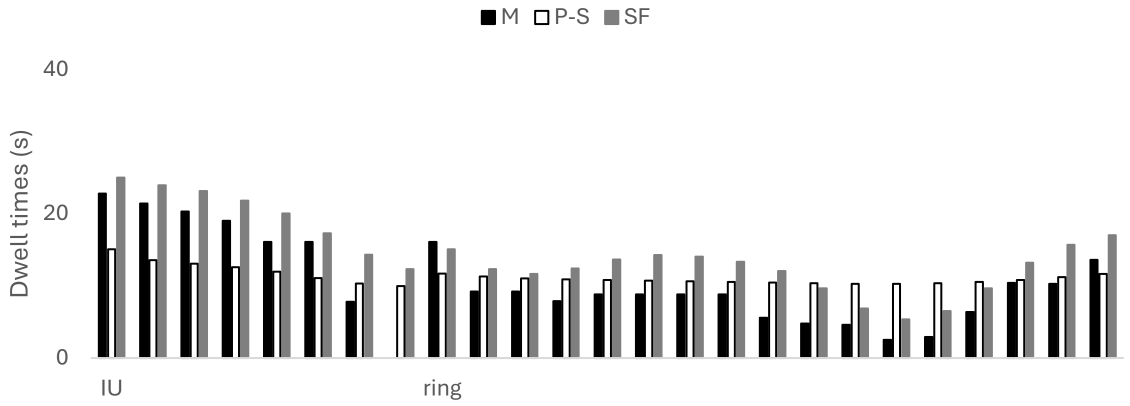

a)

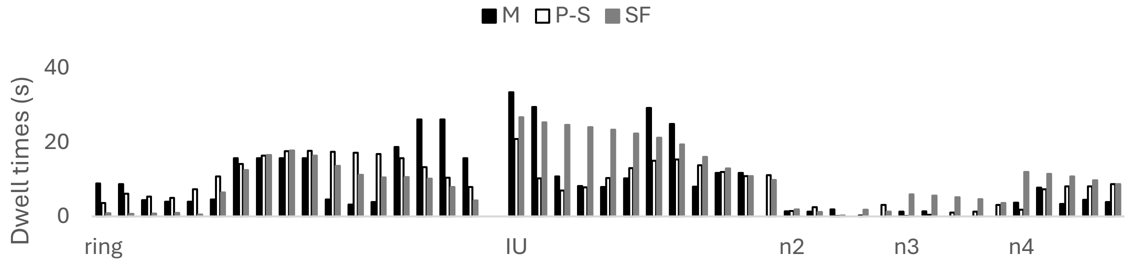

b)

Figure S9: All dwell times from the tip of the applicator part going towards the afterloader for two patients in the alternative TPS: a) IC, and b) ICIS. Here the three methods are denoted: M – manual, P-S – pseudo-structure, and SF – straightforward.

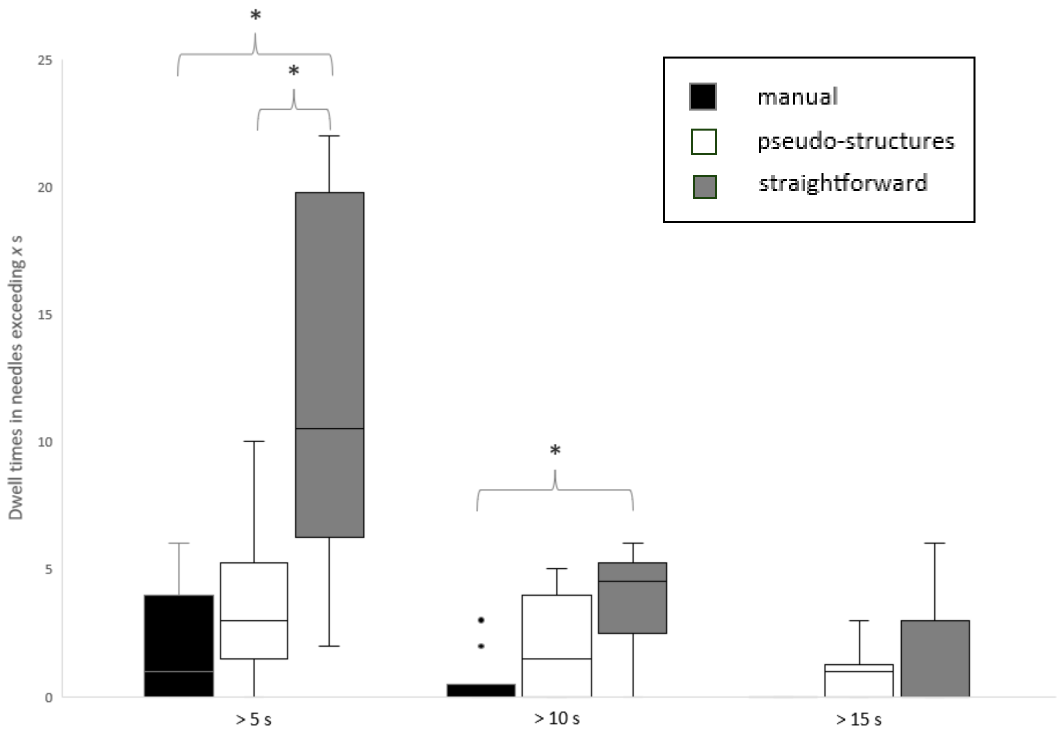

Figure S9: The number of dwell times exceeding 5 s, 10 s and 15 s in the needles for the alternative TPS. The line marks the median, the boxes span between the lower quartile value and the upper, the whiskers mark the minimum data value and the maximum, and the points are the outliers. The comparisons marked with * are significantly different using Friedman's test.

Table S1: Plan comparison data, the average for 16 treatment plans, except for relative TRAK contribution where (IC (6 treatment plans) and ICIS (10 treatment plans) were evaluated separately, were presented ± one standard deviation. The median and the range (in parenthesis) were presented and Friedmans test was used except for TRAK contribution and the sparing factors where the average ± one standard deviation and oneway ANOVA repeated measurements was used.

	**Manual**	**PS**	**SF**	**p-value Manual vs PS**	**p-value Manual vs SF**	**p-value PS vs SF**
CTV D90% (Gy)	7.6 (2.4)	7.7 (1.1)	8.0 (3.8)	n.s.	p < 0.01	p < 0.01
CTV D98% (Gy)	6.4 (4.7)	6.4 (3.2)	6.5 (4.3)	n.s.	0.04	0.07
GTV D98% (Gy)	8.0 (7.2)	8.2 (6.3)	8.3 (7.1)	n.s.	p < 0.01	n.s.
Rectum D2cc (Gy)	4.7 (2.9)	5.0 (2.5)	5.1 (2.4)	n.s.	n.s.	n.s.
Bladder D2cc (Gy)	5.3 (3.3)	5.7 (3.6)	5.8 (4.2	p < 0.01	p < 0.01	n.s.
Bowel D2cc (Gy)	4.3 (4.5)	4.4 (4.8)	5.3 (4.7)	n.s.	n.s.	n.s.
Sigmoid D2cc (Gy)	4.3 (4.6)	4.3 (5.2)	5.4 (4.6)	n.s.	p < 0.01	n.s.
Max dwell time (s)	30 (37)	35 (42)	27 (37)	n.s.	n.s.	n.s.
TRAK (cGy at 1m)	4.4 (3.2)	4.3 (3.5)	4.5 (3.7)	n.s.	p < 0.01	p < 0.01
TRAK contribution vaginal (%)* IC	55±13	63±9	53±16	0.03	n.s.	0.09
TRAK contribution intra uterine (%)* IC	45±13	39±9	47±16	0.03	n.s.	0.09
TRAK contribution vaginal (%)* ICIS	39±8	40±9	25±4	n.s.	p < 0.01	p < 0.01
TRAK contribution intra uterine (%)* ICIS	47±8	45±10	38±15	n.s.	n.s.	n.s.
TRAK contribution needles (%)* ICIS	13±8	15±9	37±16	n.s.	p < 0.01	p < 0.01
Sparing factor$_{rectum}$	0.60±0.13	0.63±0.11	0.59±0.10	n.s	n.s.	n.s.
Sparing factor$_{bladder}$	0.67±0.12	0.71±0.11	0.67±0.14	0.03	n.s.	0.01
Sparing factor$_{bowel}$	0.54±0.17	0.54±0.17	0.60±0.17	n.s.	n.s.	n.s.
Sparing factor$_{sigmoid}$	0.56±0.19	0.57±0.20	0.60±0.18	n.s.	n.s.	n.s.

PS – inverse pseudo-structure, and SF – inverse straightforward. TRAK – Total reference air-kerma. IC – intracavitary, ICIS – combined intracavitary and interstitial.
* Relative TRAK contribution from the different parts of the IC and ICIS
n.s.- not significant (p > 0.1)

References

[1] Trnková P, Baltas D, Karabis A, Stock M, Dimopoulos J, Georg D, et al. A detailed dosimetric comparison between manual and inverse plans in HDR intracavitary/interstitial cervical cancer brachytherapy. Journal of contemporary brachytherapy. 2010;2:163-70. https://doi.org/10.5114/jcb.2010.19497
[2] Sharma M, Fields EC, Todor DA. A novel two-step optimization method for tandem and ovoid high-dose-rate brachytherapy treatment for locally advanced cervical cancer. Brachytherapy. 2015;14:613-8. https://doi.org/10.1016/j.brachy.2015.05.003
[3] Tanderup K, Nesvacil N, Kirchheiner K, Serban M, Spampinato S, Jensen NBK, et al. Evidence-Based Dose Planning Aims and Dose Prescription in Image-Guided Brachytherapy Combined With Radiochemotherapy in Locally Advanced Cervical Cancer. Semin Radiat Oncol. 2020;30:311-27. https://doi.org/10.1016/j.semradonc.2020.05.008
[4] Bélanger C, Aubin S, Lavallée M-C, Beaulieu L. Simultaneous catheter and multicriteria optimization for HDR cervical cancer brachytherapy with a complex intracavity/interstitial applicator. Medical Physics. 2024;51:2128-43. https://doi.org/10.1002/mp.16874
